# All-fibre heterogeneously-integrated frequency comb generation using silicon core fibre

**DOI:** 10.1038/s41467-022-31637-1

**Published:** 2022-07-09

**Authors:** Ronit Sohanpal, Haonan Ren, Li Shen, Callum Deakin, Alexander M. Heidt, Thomas W. Hawkins, John Ballato, Ursula J. Gibson, Anna C. Peacock, Zhixin Liu

**Affiliations:** 1 Optical Networks Group, Department of Electronic and Electrical Engineering, University College London, London, UK; 2 School of Optoelectronic Engineering and Instrumentation Science, Dalian University of Technology, Dalian, China; 3 Optoelectronics Research Centre, University of Southampton, Southampton, UK; 4 Wuhan National Laboratory for Optoelectronics and School of Optical and Electronic Information, Huazhong University of Science and Technology, Wuhan, China; 5 Institute of Applied Physics, University of Bern, Bern, Switzerland; 6 Center for Optical Materials Science and Engineering Technologies, and the Department of Materials Science and Engineering, Clemson University, Clemson, SC USA

**Keywords:** Fibre optics and optical communications, Electrical and electronic engineering, Frequency combs, Optical materials and structures

## Abstract

Originally developed for metrology, optical frequency combs are becoming increasingly pervasive in a wider range of research topics including optical communications, spectroscopy, and radio or microwave signal processing. However, application demands in these fields can be more challenging as they require compact sources with a high tolerance to temperature variations that are capable of delivering flat comb spectra, high power per tone, narrow linewidth and high optical signal-to-noise ratio. This work reports the generation of a flat, high power frequency comb in the telecom band using a 17 mm fully-integrated silicon core fibre as a parametric mixer. Our all-fibre, cavity-free source combines the material benefits of planar waveguide structures with the advantageous properties of fibre platforms to achieve a 30 nm bandwidth comb source containing 143 tones with <3 kHz linewidth, 12 dB flatness, and >30 dB OSNR over the entire spectral region.

## Abstract (web summary)

Silicon core fibre is a highly nonlinear waveguide that combines the benefits of both fibre and planar waveguide systems. Here, the authors demonstrate frequency comb generation using a fully-fibre integrated silicon core fibre as a nonlinear mixer.

## Introduction

Optical frequency combs (OFCs) generate precisely-spaced phase-coherent spectral tones and have revolutionised frequency control and metrology^1^. Over the past decade, numerous developments have been focused on comb applications in optical communications, spectroscopy, microwave photonics, and optical-assisted signal processing^2–5^. Unlike metrology, in which broad ‘over-one-octave’ spectral bandwidths are the predominate requirement to enable self-referencing, communications and signal processing applications typically demand only tens of nanometers of bandwidth, but require high power, high optical signal to noise ratios (OSNR), a flat spectral response and narrow comb linewidths. In addition, compactness and temperature stability are essential prerequisites for most practical applications.

Significant efforts have been made to develop OFCs using both integrated photonic waveguides and optical fibre platforms^6–9^. Much of the work using integrated platforms has focused on Kerr soliton comb formation using dispersion-engineered microcavities, which have resulted in comb spectra spanning more than 100 nm from a single chip^10,11^. Such Kerr combs have been successfully employed in a variety of different applications, such as metrology^12^, spectroscopy^13^, frequency synthesis^14^ and in coherent communication systems, achieving 50 Tbit/s data rates using 179 individual optical carriers^15^. Despite these significant achievements, microcombs are limited by their hyperbolic secant (sech^2^) spectral power envelope, which means the comb bandwidths must greatly exceed the required operating bandwidth (i.e., C+L telecom bands for communications applications) to avoid having to use the low power wings, limiting the maximum output power-per-tone and the OSNR. Dark pulse Kerr combs with narrower spectral bandwidths (~35 nm) can offer an improvement to the power and OSNR^16^, but the need for a high quality factor cavity implies an inherent sensitivity to temperature variation. Of note is that temperature sensitivity is also an issue with other cavity-based comb sources, such as those generated using integrated mode-locked lasers^17^.

In contrast, OFCs generated via cavity-free configurations offer more flexibility in terms of spectral shaping and are in general much more robust to environmental perturbations. Such OFCs can be realised by using cascaded modulators or by pumping a highly nonlinear waveguide using high peak-power pulses, or a combination of both approaches^18,19^. For example, planar waveguides based on chalcogenide glasses and III–V semiconductor materials (e.g., AlGaAs) possess large nonlinearities and small dimensions, making them an ideal choice for compact parametric mixing stages^20,21^. However, such high index planar structures suffer from restrictive coupling requirements when compared to fibre systems as a result of their small dimensions and rectangular cross-section^22^.

In comparison, cavity-free OFCs constructed using fibre-optic platforms as the nonlinear media have the advantage of low losses and high power handling, thus significantly increasing the power-per-tone and OSNR. By integrating fibre amplifiers, fibre pulse compressors, highly nonlinear fibre (HNLF)-based saturable absorbers and parametric mixers, all-fibre OFCs have been demonstrated with bandwidths over 100 nm^23^,~0 dBm per tone and a spectral flatness of 3 dB^8^, offering superior performance for data transmission^24,25^ and microwave signal processing^26^, as well as being immediately compatible with much of the existing fibre infrastructure. However, current fibre-based OFCs are bulky due to the hundreds of meters of HNLF required to achieve significant parametric gain. Moreover, efficient parametric mixing requires careful dispersion-management over the entire HNLF length, and specifically a stable zero dispersion wavelength^27^, which increases fabrication difficulties and cost.

A long-standing goal has been to combine the materials benefits of the integrated planar waveguide structures with the advantageous waveguiding properties of fibre platforms to achieve all of the desired technical features simultaneously. The emerging class of silicon core fibres (SCF) offer a promising solution to this objective, in which a crystalline silicon core material is embedded within a conventional silica glass cladding. Compared to fibres with glassy core materials, the SCF platform offers a significantly enhanced nonlinear coefficient *γ* due to the combined effects of the high nonlinear refractive index *n*_2_ of silicon and the high core-cladding index contrast. For example, SCFs with core diameters of only a few μm can have an effective nonlinear coefficient of more than three orders of magnitude higher than that of commercially available Ge-doped HNLFs, which enables the reduction of the parametric mixer length from hundreds of meters to a few millimeters^28^. Such compact SCFs have already shown great promise for efficient nonlinear processing of signals across the extended telecoms band^29^. However, to date, the complete integration of SCFs with silica fibres has been a significant hurdle and, thus far, reported SCF results have been limited to either free space coupling^30^ or partial fibre integration^31^, which makes the coupling of dense multi-wavelength systems more challenging.

In this work, we present an all-fibre integrated SCF parametric mixer for OFC generation. Specifically, the SCF has been processed to allow for direct splicing to standard single-mode fibre (SSMF) connectors at both ends, allowing for straightforward integration with conventional fibre systems. The resulting comb structure has a bandwidth of 30 nm, with a flatness of 12 dB, >30 dB OSNR and <3 kHz linewidth, properties that are desirable for many practical applications in areas such as phase-coherent communications and micrometer/millimeter wave generation^32^. Moreover, our cavity-free design allows for tunable wavelength and comb spacing, thus enabling high resolution dual-comb spectroscopy^33^ as well as dual-comb RF processing^34^.

## Results

### SiO_2_-Si core fibre integration

The SCFs used in this work were fabricated via the molten core drawing (MCD) technique^35^. The MCD method is the most practical of the SCF production approaches as it can rapidly produce long lengths of fibre that are compatible with traditional fibre post-processing procedures. The as-drawn MCD SCFs possess a polycrystalline silicon core with typical core/cladding diameters of ~12 μm/125 μm, as detailed in the Methods. To transform the SCFs into low loss nonlinear parametric mixers that are robust and user-friendly, a multi-step tapering and splicing approach is employed. These post-processing steps are important to enhance the efficiency of the nonlinear processing in the SCFs for a number of key reasons. First, the tapering process is used both to reduce the core size as well as to improve the crystalline quality, and hence, the optical transmission^36^. This results in a SCF with core/cladding diameters of ~5 μm/125 μm and a transmission loss of 3 dB/cm. Second, a modified taper method is applied to further reduce the core size and fabricate nano-spike couplers on the end facets of the fibre. The role of the couplers is to better match the modes of the SCFs with those of the standard single-mode fibres (SSMFs), as well as to suppress reflections at the SCF/SSMF interface when splicing.

**Fig. 1:**
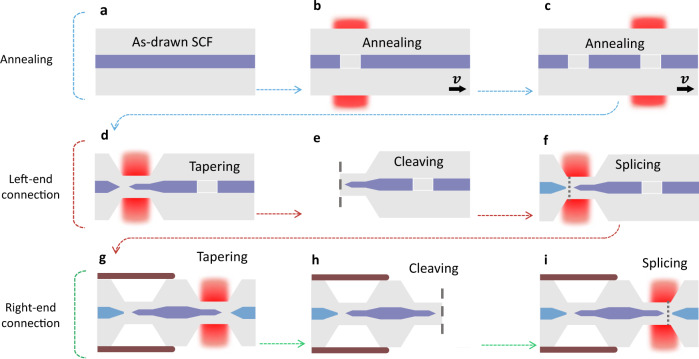
Fabrication process flow of the all-fibre heterogeneously-integrated parametric mixer. **a** Tapered SCF with core/cladding diameters of about 5 μm/125 μm. **b** Heating and tapering process to make the first void gap in the fibre core. **c** Heating and tapering to make the second void gap in the fibre core. **d** Fibre tapering process to scale down core size and collapse the void gap to form the nano-spike coupler. **e** Cleave at the center of the void to remove one side of the taper. **f** Splice the SCF nanospike to a tapered SSMF. **g** Employ a polymer tube to mechanically support the SSMF-SCF connection, keeping the fibre straight and tapering the other end of the SCF. **h** Cleave the other end and (**i**) splice to another tapered SSMF.

The full process to fabricate the integrated SCF mixer is illustrated in Fig. 1. In the first step, the SCF is heated gently while a small tension is applied along the fibre axis. Owing to the tensile stress in the as-drawn SCF, as the heated core cools and recrystallises, a void-gap can form around the heat zone (Fig. 1b). Repeating this process produces another void-gap at the other end of the fibre (Fig. 1c). Subsequently, a single sweep tapering process is used to reduce the local core/cladding ratio from 5/125 μm to 1.1/27 μm over the left-hand side of the fibre, during which the first void-gap collapses to form a nano-spike with a length of ~200 μm at the core facet (Fig. 1d). The SCF is then precisely cleaved in the core-less region and spliced with a pre-prepared tapered SSMF with same cladding diameter (Fig. 1e–f). To allow for a second connection to be made at the other end, we enclose the spliced SSMF-SCF section into a polymer capillary that mechanically supports the nano-spike coupler before applying the same procedure to the right-hand side of the fibre (Fig. 1g–i). The result is a fully integrated SCF fibre device with a total length of ~17 mm, which contains two small core sections of 1.1 μm diameter at each end (lengths of 5.1 mm and 9.1 mm at the input and output, respectively), connected by a short 3 mm length with a 5 μm diameter in the middle. Importantly, thanks to the silica cladding of the SCFs, all of these processing steps can be conducted using a single glass processing system, enabling high yield production of these heterogeneous fibre devices. The loss in the processed SCF section was determined to be ~2 dB/cm with an effective nonlinear coefficient (*γ*) of ~30 W^−1^ m^−1^. From the transmission losses, the coupling losses for the sample used in this work was estimated to be ~8 dB per facet, resulting in an end-to-end loss of ~19 dB. This fairly substantial insertion loss can be attributed to the losses associated with splicing the tapered fibres (27 μm diameter cladding) and the mode mismatch between the tapered SSMF and the nanospike. Simulations indicate that these losses could be reduced to 2 dB by optimising the cladding diameters at the connection point (<10 μm) to reduce the mismatch^37^, though these diameters are currently smaller than what our processing system can handle (see Supplementary Fig. 4).

### SCF-based comb generation

**Fig. 2:**
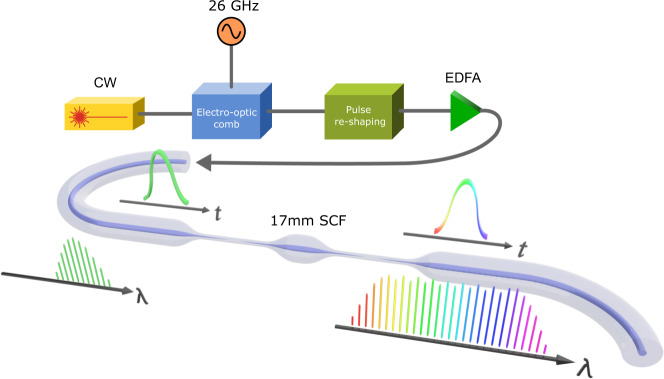
Fully integrated SCF-based parametric comb generation scheme. An electro-optic frequency comb is generated to act as a seed source for the parametric mixing stage. A pulse re-shaping stage compresses the comb pulse train to maximise the pulse peak power and enhance the parametric mixing efficiency. The optical output is then amplified and launched into a 17 mm sample of fully-integrated SCF, where parametric nonlinearities cause comb broadening. Both ends of the SCF are tapered and spliced to tapered single-mode fibre, with nano-spike couplers to facilitate coupling between the heterogenous fibre cores.

**Fig. 3:**
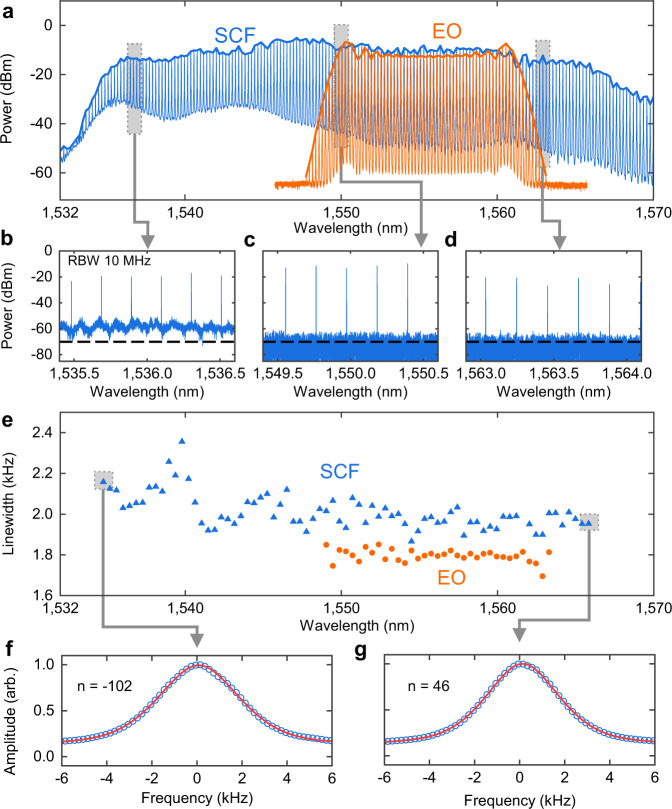
C-band parametric frequency comb generation using SCF. **a** An electro-optic frequency comb with 26 GHz line spacing (orange) was temporally reshaped and launched into a 17 mm sample of SCF at an average power of 32 dBm. The optical spectrum of the broadened parametric comb (blue) was obtained at a resolution of 0.02 nm, achieving 143 tones across a 30.0 nm bandwidth. A 10 MHz resolution BOSA was used to obtain close-in traces at different points within the comb bandwidth (**b**–**d**). The dashed line shows the instrument noise floor. Results in (**c**, **d**) are both limited by the instrument noise floor. **e** The linewidth of the parametric comb was measured using the delayed self-heterodyne interferometer (DSHI) method with a 80 km delay line and pseudo-Voigt profile fitting. The pseudo-Voigt fitting curves are shown for the two measured beat notes at the extremities of the SCF comb bandwidth (**f**, **g**).

The SCF-based parametric comb generator consists of three main sections (Fig. 2): an electro-optic (EO) comb, a fibre pulse compressor, and the all-fibre-integrated SCF device as a parametric mixer^38^. To achieve narrow linewidths, we employed a continuous wave (CW) fibre laser with ~1.6 kHz linewidth as the seed to which a phase and intensity modulation were applied to create an 11.8 nm-wide EO comb (56 tones with 26 GHz spacing), shown in orange in Fig. 3a. In the time domain, this corresponds to a pulse train with a repetition rate of 26 GHz, with each pulse exhibiting a quasi-linear frequency chirp^39^. To optimise the parametric mixing efficiency, we compressed the pulses linearly using a short length of SSMF to eliminate the frequency chirp across the center of the pulse, resulting in a full-width half-maximum (FWHM) pulse width of 610 fs. As obtaining an OFC with good spectral flatness is a key aim of this work, we implemented a nonlinear optical loop mirror (NOLM) to suppress the low-power pedestals that result from the dispersive chirp compression, which can cause significant spectral rippling after the mixing stage and reduce the mixing efficiency^8^. This also reduces the pulse FWHM down to ~440 fs before amplification to an average power of 32 dBm using a dispersion-flattened short-pulse fibre amplifier. The amplifier introduces no observable broadening of the seed pulses. The amplified pulses were subsequently launched into the 17 mm all-fibre-integrated SCF device.

The blue curve in Fig. 3a shows the spectrum of the SCF mixer output, indicating 143 tones within a bandwidth of 30 nm with a flatness of 12 dB, from 1535 nm to 1565 nm. For the estimation of the OSNR we measure the close-up spectra at 1545, 1555, and 1563 nm in Fig. 3b–d with 10 MHz resolution. Considering 0.1 nm noise bandwidth, the OSNR at the center (1550 nm) and long wavelength edge (1563 nm) of the SCF comb is >35 dB and the OSNR at the short-wavelength edge (1536 nm) is ~30 dB. The increased noise in the 1535–1545 nm region is primarily attributed to the amplified spontaneous emission (ASE) noise generated by the Er/Yb-doped fibre amplifier before the parametric amplification stage. This could be eliminated using a bandpass filter to ensure a high OSNR across the whole comb bandwidth. These results, alongside numerical analysis of the system (see Methods), show that the nonlinear spectral broadening of the comb in the SCF is dominated by self-phase modulation (SPM). As a result, the asymmetric temporal pulse shape after the NOLM leads to the observed asymmetric expansion of the comb towards shorter wavelengths (Supplementary Video 1). While efficient four-wave mixing is also achievable in the SCF mixer due to appropriate engineering of the small positive *β*_2_ and negative *β*_4_ dispersion parameters (Supplementary Fig. 1), this was not significant compared to SPM. We attribute this to the additional attenuation caused by the splice losses and free carrier effects.

Figure 3e shows the measured linewidth of the SCF comb tones (triangle marker), and the seed EO comb (circle marker), measured using the delayed self-heterodyne interferometer method^40^ for every second comb line. Typically, the EO comb linewidth increases linearly as a function of the absolute comb tone index ∣*n*∣ due to the scaling noise contribution of the RF-induced phase noise^41^. Our comb design uses an ultra-low phase noise RF signal generator (<7 fs integrated jitter from 100 Hz to 100 MHz), which results in a negligible scaling of the EO comb bandwidth with the tone index (orange circles in Fig. 3e).

After the SCF, the EO comb linewidth increases to 1.9–2.4 kHz, which we attribute to the Gordon–Mollenauer effect (nonlinearity-induced amplitude-to-phase noise conversion)^42^. This effect causes any existing amplitude noise of the comb tones (e.g., ASE noise from the EDFAs) to be converted into additional phase noise, resulting in a degradation of the spectral coherence and increased linewidths. Numerical analysis of our system shows that the spectral coherence of the SCF comb degrades as the amplifier noise figure increases, which is in agreement with the Gordon–Mollenauer effect (Supplementary Fig. 3). Since the asymmetric input pulse shape causes asymmetrical broadening of the comb, this results in greater nonlinear phase noise contributions (and thus increased comb linewidths) to be observed on the short-wavelength edge where the broadening was most significant. Nevertheless, it is clear that the SCF comb retains a well-preserved linewidth performance across the whole spectrum. Figure 3f and g show the measured beat note and their fitting using a pseudo-Voigt profile at both extremities of the comb for the linewidth characterisation. We use a pseudo-Voigt profile to account for the fact that the frequency noise of the SCF tones is a mixed contribution of 1/f (flicker) frequency noise and white frequency noise from the fibre laser^43,44^.

**Fig. 4:**
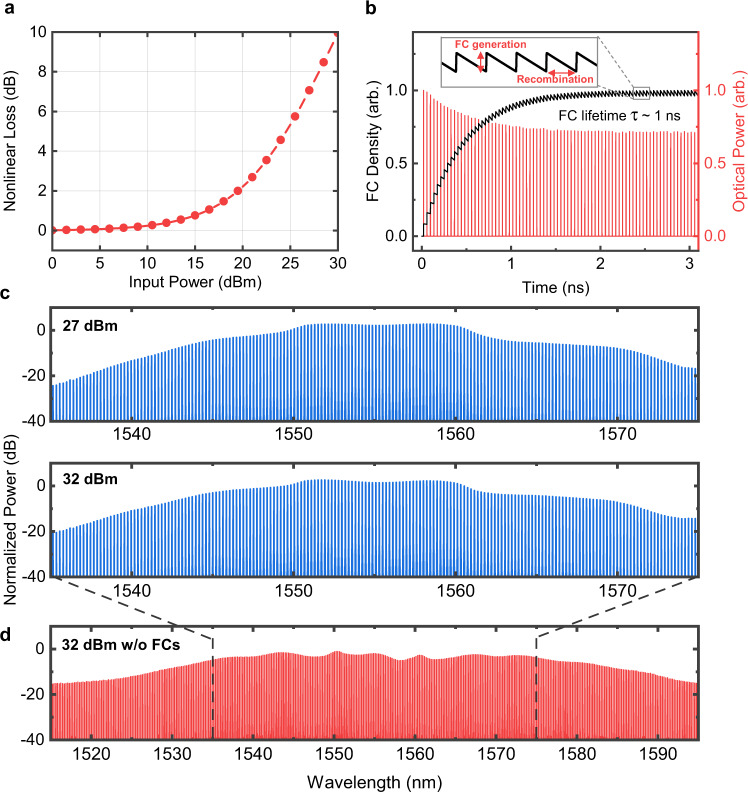
Parametric frequency comb simulation including the effects of two-photon absorption and free carriers within the SCF. **a** Nonlinear loss induced by TPA and FCs as a function of average input power. **b** Free carrier density in the silicon core versus time (black), and the optical pulse train power versus time (red). Subsequent pulses in the 26 GHz pulse train increase the free carrier density before complete recombination can occur, reaching a steady state over several nanoseconds. **c** Simulated optical spectrum at the output of the SCF mixer, at 27 dBm and 32 dBm input power (top and middle respectively) and (**d**) at 32 dBm without free carriers. TPA is included in all three spectra, and insertion loss is neglected.

To study the role of the integrated SCF device losses on the spectral bandwidth of the SCF-based comb, we simulate the comb generation system to mirror the experimental setup. The numerical simulation uses a modified generalised nonlinear Schrödinger equation (GNLSE) that incorporates two-photon absorption (TPA), free carrier absorption (FCA) and free carrier dispersion (FCD)^45^ (see Methods). We begin by evaluating the nonlinear loss due to TPA and FCA for different average launch powers within the SCF device used in our system. In these investigations, we assume zero insertion loss so that we can gauge the full potential of our approach. Figure 4a shows that the nonlinear loss becomes >1 dB when the average power increases to >17 dBm and increases exponentially thereafter.

Compared to previous studies of SCF-based nonlinear signal processing which pump SCF samples with MHz-rate pulsed sources^29^, our 26 GHz-spaced frequency comb has two orders-of-magnitude higher repetition rate. As a result, there is only a partial recombination of free carriers within each pulse period, leading to an accumulation of the free carrier density before reaching a steady state after about 2 ns, as shown in Fig. 4b. The reduction in the pulse train power due to FCA is shown as the red lines in Fig. 4b.

As expected, TPA, FCA and FCD have negative effects for wide-band comb generation and result in a reduced parametric gain, limiting the comb bandwidth at high average pump powers. As shown in Fig. 4c, increasing the average input power from 27 dBm to 32 dBm results in a negligible change of the comb spectral bandwidth due to the increase in nonlinear absorption. Moreover, the results for 27 dBm of pump power have produced a 30 nm comb bandwidth with a spectral flatness of 13 dB at the telecom C-band, similar to the experimentally measured results. The variation between the measured spectra and the simulation is only 1 dB spectral flatness for the same bandwidth, showing good agreement between the two. Thus these findings suggest that the main limitation to the comb bandwidth is the free carrier effects, rather than the insertion loss, and that the ‘sweet spot’ observed in both our simulations and experiment is a balance between the input power and nonlinear losses. Nevertheless, OFCs with a 30 nm bandwidth are suitable for many target applications in communications and signal processing, and the SCF comb delivers both high power-per-tone and all-fibre connectivity required for practical systems.

## Discussion

The last 20 years of frequency comb development has resulted in an array of comb generation technologies that have been used in numerous electronic and photonic applications. Yet, despite the wide array of platform options, there are relatively few OFCs designed specifically with the telecommunications and optical signal processing applications in mind. Our SCF-based OFC fills an important gap in the frequency comb family to provide a cavity-free, temperature-insensitive, flat and high power OFC source with a compact and portable form. The SCF-based OFC is able to achieve comparable performance to other all-fibre nonlinearly broadened frequency combs in literature while using orders of magnitude shorter mixer lengths, providing a practical alternative to conventional HNLFs (Supplementary Table 2). Although the bandwidth of our system is currently limited by the free carrier effects associated with the silicon core, it is possible to mitigate these effects using carrier sweep-out schemes employed in planar silicon systems^46^. This could potentially be achieved by introducing two platinum rods next to the semiconductor core when making the fibre preform^47^. Alternative schemes involving gold-doping of the crystalline Si core can also be implemented to reduce the free-carrier lifetime^48^.

Removing FCA would result in further increase of the bandwidth to >65 nm, as illustrated by the simulated spectrum in Fig. 4d. In the future, the connection losses between the SSMF-SCF could also be reduced to below 1 dB per facet by optimising the nano-spike coupler design (e.g., employing a thinner silica cladding with outer diameter <10 μm)^37^, though this would require a customised mounting rig during the splicing, which is not currently available (Supplementary Fig. 4). With the reduced SSMF-SCF connection losses, we envisage replacing the HNLF in the NOLM stage with another section of SCF to reduce the size of the OFCs and enable a more compact SCF comb solution.

One of the areas in which the all-fibre SCF mixer shows significant promise is in dual-comb applications. It has been previously demonstrated that the phase noise of dual EO frequency combs is highly coherent^49^. However, this phase coherence may degrade when using conventional HNLF for nonlinear broadening due to the relatively long fibre lengths required (typically 100s of metres). The all-fibre SCF mixer only requires 10–20 mm fibre length for bandwidth expansion, minimising the group delay walk off between the dual combs and preserving the coherence between both combs.

In summary, we have presented heterogeneous integration of a SCF with SSMFs for compact and efficient all-fibre frequency comb generation. Using our fabricated SCF as a mixer, we obtain 143 tones in a flat, 30 nm bandwidth frequency comb that exhibits narrow linewidths across the whole frequency region. Our approach harnesses the merits of nonlinear silicon waveguides and optical fibre platforms, underpinning comb applications requiring signal generation, processing and detection.

## Methods

### SCF fabrication

The as-drawn SCFs are fabricated using the molten core fibre drawing (MCD) technique. This process uses a standard fibre drawing tower to heat and melt the silicon core that is surrounded by a softened silica cladding (drawing temperature of 1950 °C), which acts as a crucible to retain the fibre profile as it is drawn down, as detailed in ref. ^50^. A thin layer of calcium oxide is included as an interfacial barrier between the core and cladding during the drawing process, which limits dissolution of silica from the cladding into the silicon core and reduces the thermal strain arising from high-temperature processing. The as-drawn SCFs have a poly-crystalline core material with uniform core/cladding diameters of 12 μm/125 μm. To improve the crystalline quality and reduce the losses of the as-drawn fibres, we insert the original SCFs into a silica capillary (400 μm/150 μm inner/outer diameter) and taper this down to have core/cladding diameters of about 5 μm/125 μm. The fabrication is realised using a glass processing system (Vytran GPX-3400-V4), which is widely accessible for heat-polishing, tapering and splicing.

Similar to the nano-taper couplers commonly used in planar silicon waveguides^51^, nano-spike couplers are fabricated on the SCF facets to improve the coupling to SSMF. The nano-spikes are created by carefully tapering the SCF with the prepared void-gap, which occurs as a result of releasing the tension in the SCFs that is built-in due to the thermal expansion mismatch of the core/cladding materials. Splicing of the tapered SCF with nano-spike couplers on both ends of the tapered SSMFs is achieved by applying a heating power of 63 W over 7 s.

### Seed comb generation

Our seed comb begins with modulating a 1555.72 nm CW signal from a fibre laser using a LiNbO_3_ Mach–Zehnder modulator and two phase modulators (Supplementary Fig. 2). The 1.6 kHz-linewidth CW source was amplified to 33 dBm by a polarisation-maintaining fibre amplifier before launching into the modulators. The modulators transform the CW light into a repeated pulse train with the pulse period corresponding to each modulation cycle^52^. The resulting linear chirp yields pulses with relatively flat spectral envelopes for a tone spacing of 26 GHz. A low phase noise RF source was employed to generate the 26 GHz signal that drives the modulators. Generally, an arbitrary frequency can be used to enable a tunable tone spacing that suits DWDM applications. In our system, the RF frequency was tunable between 22 and 26.5 GHz, limited by our frequency synthesiser and the electronic devices.

Subsequent linear pulse compression was realised by compensating the spectral phase of the pulses using a reel of 65 m of SSMF, which provides second-order dispersion to compress the pulses to their Fourier-transform limit. The pulse full-width half-maximum (FWHM) was measured to be ~610 fs using an optical autocorrelator (FEMTOCHROME FR-103XL) with a Gaussian pulse profile assumed. An erbium-doped fibre amplifier (FA2) was used to amplify the pulse train before reshaping via a NOLM. The NOLM consists of a 3 dB optical coupler connected to a 105 m Ge-doped HNLF with a dispersion of −0.38 ps nm^−1^ km^−1^ and a nonlinear coefficient of >10 W^−1^ km^−1^ at 1550 nm. The fibre was placed in the NOLM loop, along with a polarisation controller and a 5 dB attenuator. The NOLM acts as an intensity discriminator, which transmits the high-power peak regions at the center of each pulse and reflects the low-power background, providing a pedestal suppression ratio of 17.1 dB and additional pulse compression^53^.

### Linewidth characterisation

The linewidth characterisation is performed by using a delayed self-heterodyne interferometer with 80 km ultra-low loss single-mode fibre, providing about 4 μm delay or ~1.2 kHz spectral resolution, necessary for characterising the narrow linewidth tones. As the seed CW source for the comb is a fibre laser, there is a significant 1/f-type (flicker) frequency noise contribution to the frequency noise power spectral density^43^. In this case a Lorentzian profile cannot be assumed for the line shape since the frequency noise power spectral density is not dominated by white frequency noise. As such, we use a pseudo-Voigt profile, rather than a Lorentzian profile, to fit the measured beat note to appropriately account for the 1/f-induced linewidth broadening^44^. While the RF driving signal also contributes both white phase (f^2^ frequency noise) and coloured phase noise to the comb noise power spectral density, this is difficult to generalise and has been neglected in this analysis.

### Simulation

We simulate the comb generation scheme with the same properties as the experiment to ensure a close match to our measured results. Pulse train propagation through the SCF was modelled by the GNLSE, including TPA and free carrier effects (FCA and FCD)^54^: [Eq. 1]

1 $$\frac{\partial E}{\partial z}=-\frac{{\alpha }_{l}}{2}E+\mathop{\sum }\limits_{m=2}^{4}i\frac{{i}^{m}{\beta }_{m}}{2!}\frac{{\partial }^{m}E}{\partial {t}^{m}}+i\gamma \left(| E{| }^{2}E+\frac{i}{{\omega }_{0}}\frac{\partial }{\partial t}(| E{| }^{2}E)\right)-\frac{\sigma }{2}(1+i\mu ){N}_{c}E$$

where *E* is the electric field envelope, *α*_*l*_ is the linear attenuation and *β*_*m*_ is the *m*-th order dispersion parameter. TPA is included as the imaginary component of the nonlinear coefficient *γ* [Eq. 2]:

2 $$\gamma =\frac{2\pi {n}_{2}}{\lambda {A}_{{{{{{{{\rm{eff}}}}}}}}}}+i\frac{{\beta }_{{{{{{{{\rm{TPA}}}}}}}}}}{2{A}_{{{{{{{{\rm{eff}}}}}}}}}}$$

where *n*_2_ is the Kerr coefficient, *β*_TPA_ is the TPA parameter and *A*_eff_ is the effective mode area. FCA and FCD are included in the last term in equation (1), where *σ* is the FCA coefficient and μ = 2*k*_*c*_*k*_0_/*σ*, with *k*_0_ = 2*π*/*λ* and *k*_*c*_ is the free-carrier-induced refractive index change. The magnitude of the FCA and FCD effects are governed by the rate equation for the free carrier density *N*_*c*_^55^: [Eq. 3]

3 $$\frac{\partial {N}_{c}(z,t)}{\partial t}=\frac{{\beta }_{{{{{{{{\rm{TPA}}}}}}}}}}{2h{v}_{0}}\frac{| E(z,t){| }^{4}}{{A}_{{{{{{{{\rm{eff}}}}}}}}}}-\frac{{N}_{c}(z,t)}{\tau }$$

where *τ* is the free carrier lifetime. The GNLSE was solved using the split-step Fourier method (SSFM). Dispersion was included up to fourth-order and Raman scattering was neglected due to the short duration of our pulses. To accurately model the tapered SCF device with a varying core diameter, the SCF was separated into three distinct segments of length 5.1 mm, 3 mm and 9.1 mm (input taper, middle and output taper respectively). These corresponded to core diameters of 1.1 μm for the small tapered regions and 5 μm for the middle region, and the mode properties of each diameter were estimated from COMSOL Multiphysics software simulations. The parameters used in the simulations are listed in Supplementary Table 1 and Fig. 1, and were obtained via a combination of the mode simulations and laboratory experiments. The free carrier density and pulse train shown in Fig. 4b was taken from the final step of the SSFM to illustrate the free carrier density reaching steady state.

## Supplementary information

Supplementary Information

Peer Review File

Description of additional supplementary files

Supplementary Video

## Data Availability

The data that support the plots within this paper and other findings of this study are deposited in the UCL Research Data Repository, 10.5522/04/20108783^56^.
