# Supplementary material for: All-fibre heterogeneously-integrated frequency comb generation using silicon core fibre
Source: Nat Commun. 2022 Jul 9;13:3992. doi: 10.1038/s41467-022-31637-1 (PMC9271068; doi:10.1038/s41467-022-31637-1)
Supplement: Supplementary file 1 — Supplementary Information [file 41467_2022_31637_MOESM1_ESM.pdf]

Supplementary Information: All-fibre heterogeneously-integrated frequency comb generation using silicon core fibre

R. Sohanpal, H. Ren, L. Shen, C. Deakin, A. M. Heidt, T. W. Hawkins, J. Ballato, U. J. Gibson, A.C. Peacock and Z. Liu

Supplementary Note 1: Simulation Parameters

The parameters used in the SCF simulation are given in Supplementary Table 1. The length of the SCF sample was measured using a standard laboratory microscope. As most of the parametric broadening occurs in the 1.1µm core region at the input and output of the SCF (corresponding to 14.2mm of the 17.2mm-long device), only the propagation parameters for this core diameter are listed here. The core diameter was estimated from microscope images, and the effective mode area and dispersion were calculated using COMSOL Multiphysics software. The transmission losses were estimated via a combination of cutback measurements using SCFs with the same core diameters. The linear loss value was confirmed during the nonlinear characterization of the SCF, where the TPA coefficient was estimated from the nonlinear saturation and the nonlinear refractive index was found by fitting the spectral broadening due to SPM [1]. The insertion loss was measured by launching a CW source through the fibre, from which the propagation loss was subtracted to obtain the total coupling loss. The coupling loss per facet was then assumed to be half of the total coupling loss and is mostly associated with the mismatch in the fibre modes. The free carrier lifetime, FCA parameter and FC-induced change in refractive index are standard values for crystalline silicon waveguides of these dimensions, obtained from literature [2]. All parameters listed were measured or estimated at 1550 nm.

Parameter	Symbol	Value
Center wavelength	λ	1555 nm
Comb repetition rate	f_m	26 GHz
Input pulse FWHM	T_{FWHM}	440 fs
SCF total length	L	1.72 cm
Linear loss	α_{linear}	2 dB/cm
Insertion loss	$\alpha_{\mathrm{insertion}}$	8.5 dB
Core diameter	d	1.1 µm
Effective mode area	a_{eff}	0.55 µm^2
Dispersion coefficient	D	-222.8 ps nm^{-1} km^{-1}
Dispersion slope	S	1.74 ps nm^{-2} km^{-1}
Kerr effect coefficient	n_2	4×10^{-18} m^2 W^{-1}
Nonlinearity coefficient	γ	29.6 W^{-1} m^{-1}
Free carrier lifetime	τ	1 ns
TPA coefficient	β_{TPA}	5×10^{-12} m W^{-1}
FCA parameter	σ	1.45×10^{-21} m^2
FC-induced change in refractive index	κ_c	1.35×10^{-27} m^3

Supplementary Table 1: **Simulation parameters used to model the silicon core fibre (SCF).**

Supplementary Note 2: SCF Dispersion

For efficient four-wave mixing to occur, the phase-matching condition must be satisfied, given by $\Delta k = 2\gamma P_{pump} - \Delta k_{linear} = 0$, where Δk is the total phase mismatch, $2\gamma P_{pump}$ is the nonlinear phase shift and Δk_{linear} is the linear phase mismatch. Close to the zero dispersion wavelength (ZDW), the linear phase mismatch can be expressed as:

$$\Delta k_{linear} = -\beta_2 \Delta\omega^2 - \frac{1}{12}\beta_4 \Delta\omega^4 \tag{1}$$

where β_2 and β_4 are the second-order and fourth-order dispersion terms respectively, and $\Delta\omega$ is the frequency offset. Given that the second-order and fourth-order terms for the SCF possess opposite signs (positive and negative respectively, as shown in Supplementary Fig. 1) the linear phase mismatch Δk_{linear} can be close to zero, giving a phase-matching region of width

$$\Delta\omega = \sqrt{\frac{12|\beta_2|}{|\beta_4|}} \tag{2}$$

which provides an enhanced FWM bandwidth for parametric expansion.

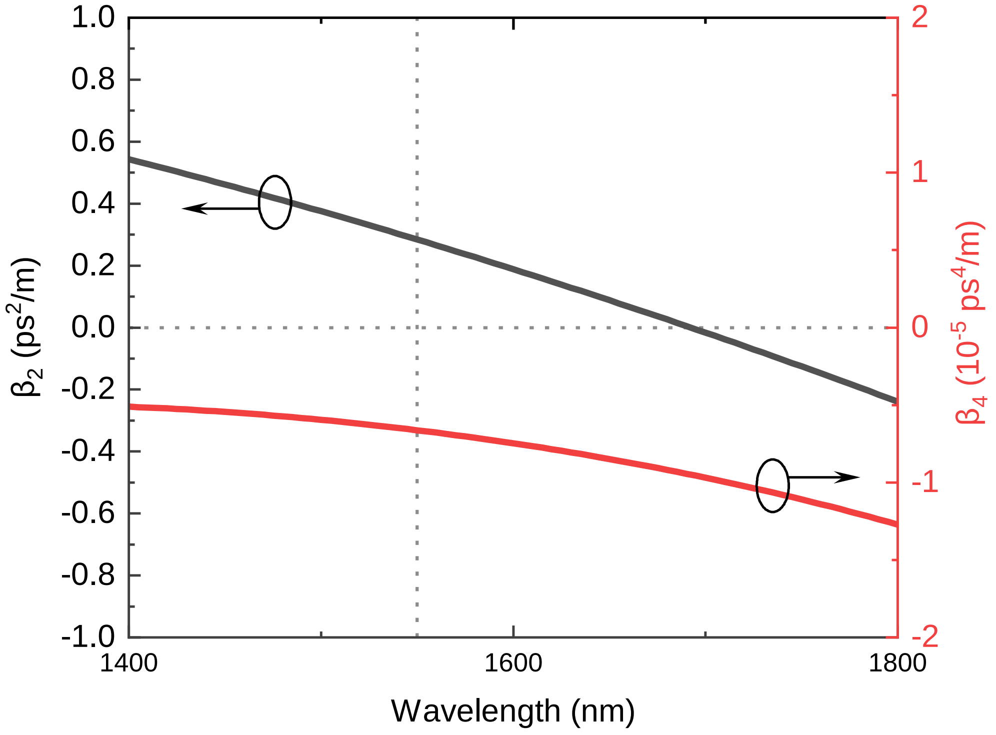

Supplementary Figure 1: **Calculated second-order (GVD) and fourth-order dispersion (FOD) versus wavelength for the SCF sample.** The vertical dashed line indicates the CW seed laser wavelength (1555 nm).

Supplementary Note 3: SCF-based Comb Generator

The comb generator setup is shown in Supplementary Fig. 2. A 5 kHz linewidth CW laser at 1555 nm seeds a cascade of one intensity modulator and two phase modulators, generating an electro-optic comb with a repetition rate of 26 GHz. The pulse train at the output of the EO comb is then linearly compressed by a 65 m length of SSMF, before it is amplified by an EDFA and launched into a nonlinear optical loop mirror (NOLM). The NOLM, consisting of a 105 m length of HNLF, a polarisation controller and a 5 dB optical attenuator, filters out the optical pedestals and further compresses the pulses. These pulses (440 fs FWHM) are then amplified again by a dispersion-flattened EDFA and launched into the SCF sample. The output is detected by an optical spectrum analyzer.

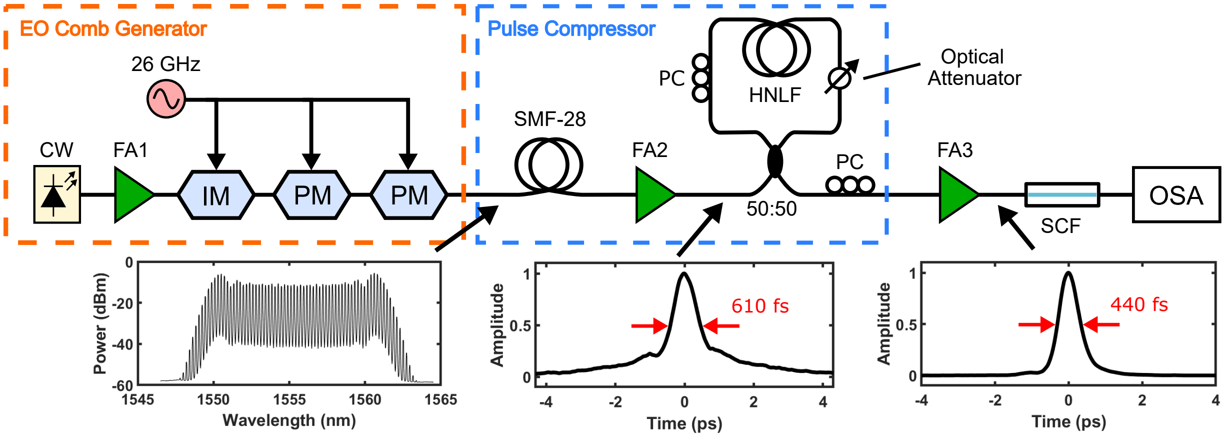

Supplementary Figure 2: **SCF-based parametric comb generation experimental setup.**

Supplementary Note 4: First-order Coherence Analysis

The modulus of the first-order coherence $|g_{12}|$ is used to evaluate the shot-to-shot fluctuations in spectral power, which reflects the overall spectral coherence of the SCF-based parametric comb. This is particularly important in applications where broadband temporal coherence is required, such as frequency metrology. The temporal coherence is also indicative of the comb tone linewidth and visibility. The first-order coherence can be defined by:

$$|g_{12}| = \left| \frac{\langle S_1^*(\lambda) S_2(\lambda) \rangle}{\sqrt{\langle |S_1(\lambda)|^2 \rangle \langle |S_2(\lambda)|^2 \rangle}} \right| \tag{3}$$

where the angle brackets reflect the ensemble averages over all pulse pairs at the output of the SCF, $[S_1(\lambda), S_2(\lambda)]$. A coherence value of close to 1 implies strong stability in amplitude and phase, whereas a value close to zero implies little to no stability.

Supplementary Fig. 3 shows the single-pulse spectra obtained at the output of the SCF for 24 independently-simulated pulses at different OSNR, generated by assuming different optical amplifier noise figures (3 dB, 11 dB and 21 dB) and their corresponding spectral coherence. It can be seen that the increase in amplifier noise figure leads to noisier wings in the SCF comb spectrum, alongside a reduction in the bandwidth over which the comb spectrum is coherent. As the coherence is related to the optical linewidth of the comb tones, the reduction in coherence in the short-wavelength region is linked to an increase in the comb linewidths, which was experimentally observed (Fig.3e in the main text). The simulations also confirms the experimental observation that the long-wavelength edge of the comb is less affected by the reduction in coherence and corresponding linewidth increase since the asymmetric temporal pulse shape after the NOLM favours nonlinear comb expansion towards shorter wavelengths.

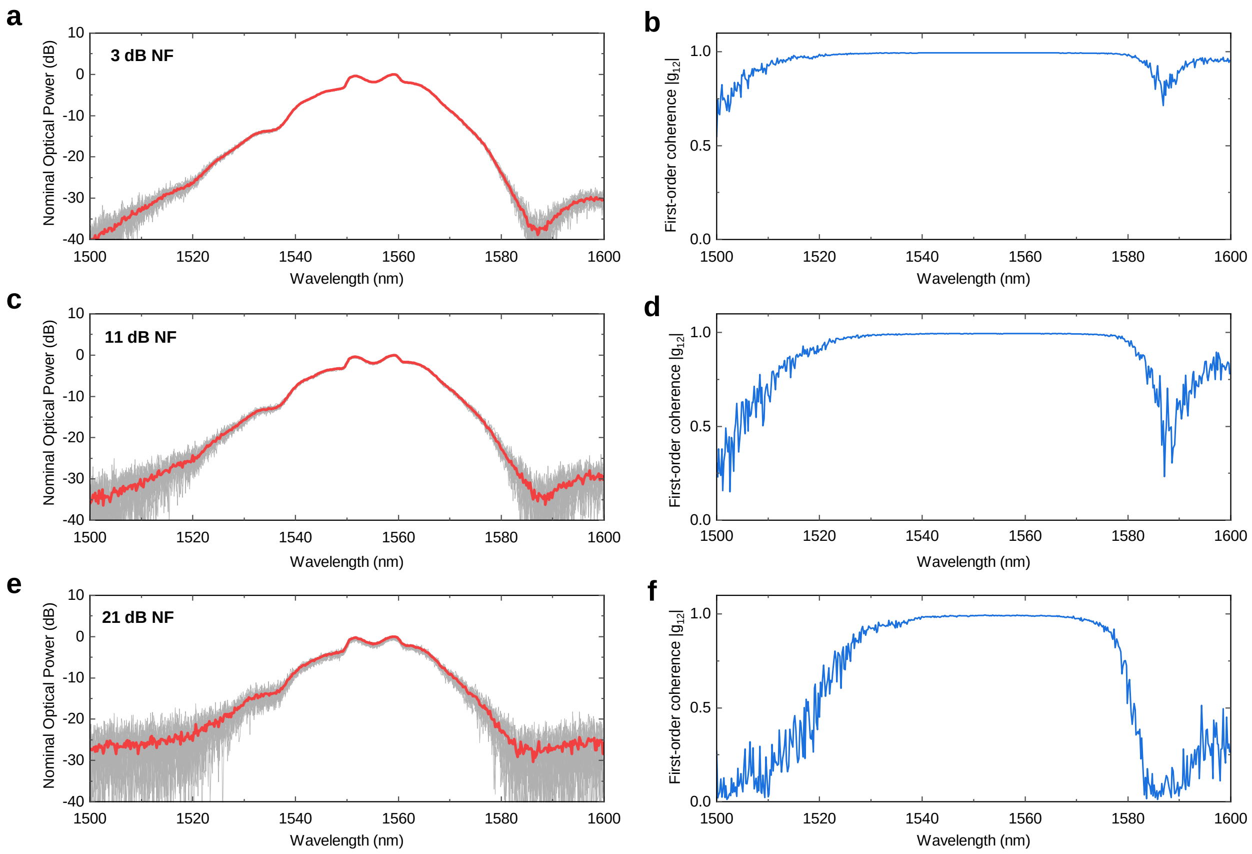

Supplementary Figure 3: **Simulated first-order coherence of the SCF-based parametric comb with different final-stage EDFA noise figures.** 24 noisy pulses at the output of the final-stage amplifier (FA3 in Supplementary Fig. 2) were propagated through the SCF sample and the spectrally-resolved modulus of the first-order coherence ($|g_{12}|$) was calculated over the comb bandwidth for zero path difference. This was repeated for amplifier noise figures of 3 dB (a-b), 11 dB (c-d) and 21 dB (e-f). The figures in the left column show individual spectra for each pulse (gray) and the mean spectrum (red). The fluctuations of the gray spectra show the noise-induced shot-to-shot variation in spectral power between pulses. The figures in the right column show the temporal coherence, showing a degradation in the coherent bandwidth of the comb when the amplifier noise increases.

Supplementary Note 5: Tapered Nanospike Coupling Loss Analysis

Supplementary Figure 4a shows the simulated coupling loss for the tapered nanospike. The simulations were conducted using COMSOL Multiphysics software at 1550 nm. The nanospike starts with a 5 nm diameter and widens to a core diameter of 1.1 µm at the end of the transition length. The light transmission can be split into 4 parts: in section I, the light is transmitted into the tapered region of the SMF, where the mode expands slowly and becomes cladding guided. The loss in this section is negligible as the fibre is adiabatically tapered. The large mode area means that the fibre is not sensitive to misalignment at splicing joint. In section II, the light enters the coreless section of the SCF. In this section, the SCF only has a thin layer of CaO that acts as the fibre core. Its refractive index is assumed to be 1.6, which is between the refractive index of silica and CaO. In section III, the light is coupled into the nanospike. In section IV the light propagates into the main SCF body.

For a CaO layer with a thickness of 260 nm in our device, coupling loss versus nanospike length for different cladding diameters is shown in Supplementary Figure 4b. Material absorption and boundary scattering were assumed to be zero to investigate only the effects of a change in spike dimension on the coupling losses. It can be seen that smaller cladding sizes and longer spike lengths lead to higher coupling efficiencies. When the device dimension is optimised, theoretically the coupling loss can be negligible small. Further analysis of the light propagation can be found in [3].

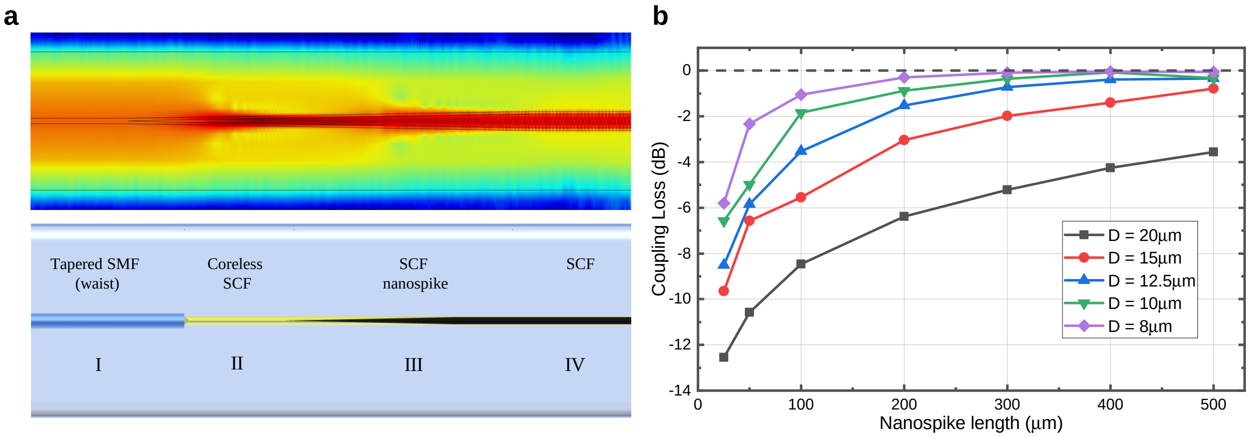

Supplementary Figure 4: **Simulated light transmission through the tapered nanospike and coupling loss analysis.** Light propagation through the tapered nanospike is shown in (a) for core/cladding diameters of 1.1 µm/10 µm. A plot of coupling loss versus nanospike length is shown in (b) for different cladding diameters.

Supplementary Note 6: Frequency Combs in Literature

Supplementary Table 2 shows a comparison between various all-fibre frequency comb generators in literature, including the SCF-based comb generator shown in this work. Note that the nonlinear medium used in all of these studies (except in this work) is doped highly nonlinear fibre (HNLF). Any additional HNLF used in these publications (e.g. for a NOLM) is excluded from the nonlinear fibre length mentioned in the table.

Reference	Launch power (dBm)	Repetition rate (GHz)	Nonlinear fibre length (m)	Bandwidth (nm)	Approx. flatness (dB)
[4]	27.7	200 & 400	349	160	10
[5]	31.8	10	1000	8	5
[6]	31.5	12.5	83	150	6
[7]	31.8	10	100	8.1	10
[8]	32.3	10	150	28	3.5
[9]	26.0	6.25	200	100	3
[10]	25.0	25	290	25	10
[11]	27.0	10	200	120	2
**This work**	**32**	**25**	**0.017**	**30**	**12**

Supplementary Table 2: **All-fibre nonlinearly broadened frequency combs in literature.**

References

[1] Ren, H. *et al.* Nonlinear optical properties of polycrystalline silicon core fibers from telecom wavelengths into the mid-infrared spectral region. *Opt. Mater. Express* **9**, 1271–1279 (2019). URL http://www.osapublishing.org/ome/abstract.cfm?URI=ome-9-3-1271.

[2] Yin, L. & Agrawal, G. P. Impact of two-photon absorption on self-phase modulation in silicon waveguides. *Opt. Lett.* **32**, 2031–2033 (2007). URL http://www.osapublishing.org/ol/abstract.cfm?URI=ol-32-14-2031.

[3] Ren, H. *et al.* Tapered silicon core fibers with nano-spikes for optical coupling via spliced silica fibers. *Opt. Express* **25**, 24157–24163 (2017). URL http://www.opticsexpress.org/abstract.cfm?URI=oe-25-20-24157.

[4] Tong, Z. *et al.* Spectral linewidth preservation in parametric frequency combs seeded by dual pumps. *Opt. Express* **20**, 17610–17619 (2012). URL http://opg.optica.org/oe/abstract.cfm?URI=oe-20-16-17610.

[5] Yang, T. *et al.* Comparison analysis of optical frequency comb generation with nonlinear effects in highly nonlinear fibers. *Opt. Express* **21**, 8508–8520 (2013). URL http://opg.optica.org/oe/abstract.cfm?URI=oe-21-7-8508.

[6] Zhang, X. *et al.* Sub-100 fs all-fiber broadband electro-optic optical frequency comb at 1.5µm. *Opt. Express* **28**, 34761–34771 (2020). URL http://opg.optica.org/oe/abstract.cfm?URI=oe-28-23-34761.

[7] Supradeepa, V. R. & Weiner, A. M. Bandwidth scaling and spectral flatness enhancement of optical frequency combs from phase-modulated continuous-wave lasers using cascaded four-wave mixing. *Opt. Lett.* **37**, 3066–3068 (2012). URL http://www.osapublishing.org/ol/abstract.cfm?URI=ol-37-15-3066.

[8] Wu, R., Torres-Company, V., Leaird, D. & Weiner, A. Supercontinuum-based 10-GHz flat-topped optical frequency comb generation. *Opt. Express* **21**, 6045–6052 (2013). URL http://opg.optica.org/oe/abstract.cfm?URI=oe-21-5-6045.

[9] Ataie, V. *et al.* Ultrahigh Count Coherent WDM Channels Transmission Using Optical Parametric Comb-Based Frequency Synthesizer. *Journal Of Lightwave Technology* **33**, 694–699 (2015).

[10] Vikram, B. *et al.* Generation of a multi-wavelength source spanning the entire C-band by nonlinear spectral broadening of dual-carrier electro-optic frequency combs. *OSA Continuum* **3**, 2185–2194 (2020). URL http://opg.optica.org/osac/abstract.cfm?URI=osac-3-8-2185.

[11] Ataie, V., Myslivets, E., Kuo, B. P.-P., Alic, N. & Radic, S. Spectrally equalized frequency comb generation in multistage parametric mixer with nonlinear pulse shaping. *Journal of Lightwave Technology* **32**, 840–846 (2014).
