# Supplementary material for: All-fibre heterogeneously-integrated frequency comb generation using silicon core fibre
Source: Nat Commun. 2022 Jul 9;13:3992. doi: 10.1038/s41467-022-31637-1 (PMC9271068; doi:10.1038/s41467-022-31637-1)
Supplement: Supplementary file 3 — Description of additional supplementary files [file 41467_2022_31637_MOESM3_ESM.pdf]

Description of additional supplementary files

Supplementary Video 1: Simulated spectrogram showing the spectral evolution of the comb output for different propagation distances through the SCF sample.
